# Towards a research agenda for global malaria elimination

**DOI:** 10.1186/1475-2875-7-S1-S1

**Published:** 2008-12-11

**Authors:** Marcel Hommel

**Affiliations:** 1 Editor-in-Chief, Malaria Journal; 2 Institut Pasteur, 28 rue du Dr Roux, Paris 75724 Cedex, France

## 

For the past twenty years, many scientific papers on malaria have begun with statements such as 'there are an estimated three billion people at risk of the disease'; or '300–600 million episodes of clinical falciparum malaria occur each year, killing between one and three million individuals annually'; or 'the global burden of malaria exceeds 40 million disability-adjusted life years' [1,2]. These figures may once have been a credible approximation, but need to be looked at today with considerable scepticism, even when they appear in official statements. Indeed, figures of malaria incidence/prevalence or of disease burden, are mostly based on reports of 'presumptive diagnosis', the accuracy of which is 50% at best and less than 5% in situations of low endemicity. Figures relating to malaria mortality are even less reliable, because the exact cause of death is always difficult to determine, even in hospital settings [3]. Recent studies of severe malaria in children in Malawi and in Kenya suggest that, in at least 25% of cases, the WHO definition of severe malaria is unable to exclude concomitant bacterial or viral infections, which may actually be responsible for the fatal outcome [4]. The field reality is worse, when one considers that many malaria cases occur in remote areas, where the resources for making adequate diagnosis are not available.

However, a general pattern is emerging, based on recent publications and public statements and despite this inadequacy of the baseline figures, that malaria incidence is decreasing worldwide and, in some cases, this has actually been documented by reliable data. For example, child mortality on the Kenyan coast has been reduced [5], and, over the past few years, there has been a fall of 50–75% in malaria incidence in Zanzibar [6], Eritrea [7], Ethiopia and Rwanda [8]. Interestingly, there have been reports from travel clinics in Europe indicating a reduction in their imported malaria cases [9,10]. Policy-makers are keen to believe that this reduction of malaria cases and of malaria mortality is the result of their activity and the mass interventions that have been made possible through unprecedented funding for malaria control. An estimated US$1.5 billion was spent in 2007, 47% of which was disbursed by international donor agencies, including the Global Fund [11]. Existing control measures, including large-scale distribution of insecticide-impregnated bed nets, improved diagnosis using rapid tests, treatment using a combination of effective drugs including artemisinin, and prevention/treatment of high-risk target groups such as pregnant women, are known to work and will undoubtedly have had an impact when implemented on an operational scale in some endemic areas. However, a decrease of malaria incidence has not always followed implementation of control measures and other factors, both human and climatic, may have also played a part. Nevertheless, the decreasing pattern of incidence is encouraging and, by giving funding agencies a first impression of success, it has helped enormously in the advocacy for further increases in the global funding effort, which are considered necessary to achieve malaria control. As a result, Bill and Melinda Gates surprised the malaria community in October 2007 by expressing their firm commitment to global '*malaria eradication' *[12]. This was a bold statement, since the word 'eradication' had not been used in official statements since the early 1970s, when the failure of an earlier global eradication attempt was recognized. The statement has had three noticeable effects: (i) almost immediately, other agencies, hitherto committed to control, jumped on the eradication band wagon, (ii) it put the goal of '*elimination*', if not yet of 'eradication', on the global agenda with a notional target date of 2025, and (iii) the notion of elimination by 2025 captured the imagination of the scientific community working on malaria, which moved in a short space of time from a realistic awareness of the enormity of the complex and seemingly insoluble problem of malaria control to cautious optimism that elimination by 2025 is a real possibility – the Emperor's New Clothes, perhaps?

Nobody believes that elimination will be easy to achieve and it will undoubtedly require sustained, high level funding and political commitment for many years. Indeed, history shows that the single most important cause of failure of the 1960s eradication campaign was the rapid loss of political commitment once control had been shown to work. The move from control to elimination is actually a quantum leap. If it were once possible to state that all the tools for control were available and that no further research was required, it is doubtful whether elimination could be achieved with those same tools. There is now a need to define a new research agenda and to identify the differences between control and elimination.

*Malaria Journal *is now in its seventh year of existence and it has become a major journal in the discipline, reporting on all aspects of malaria, 'from the bench, to the bedside and to the bush'. There will no doubt be many papers published on malaria elimination in the future, but it is fitting that *Malaria Journal *should be the first journal to publish a series of review articles looking at the many aspects of the research agenda for malaria elimination on a global scale.

## Competing interests

The author declares that they have no competing interests.
